# Hydroxyurea treatment for sickle cell disease in Nairobi informal settlements: a retrospective cohort study

**DOI:** 10.3389/fmed.2026.1889846

**Published:** 2026-08-26

**Authors:** Maria Vittoria De Vita, Matilu Mwau, Anastasia Omoding, Washington Njogu, Gianfranco Morino, Caleb Mike Mulongo, Annette Aigner, Lena Oevermann

**Affiliations:** 1 Amici del Mondo World Friends – Ruaraka Uhai Neema Hospital, Nairobi, Kenya; 2 Department for Oncology and Hematology, Charité – University Medical Center Berlin, Corporate Member of Freie Universität Berlin and Humboldt-Universität zu Berlin, Berlin, Germany; 3 Children Sickle Cell Foundation, Nairobi, Kenya; 4 Kenya Medical Research Institute, Nairobi, Kenya; 5 Gertrude's Garden Children's Hospital, World-Class Paediatric, Nairobi, Kenya; 6 Tunu Consulting Hub, Nairobi, Kenya; 7 Center for Stroke Research Berlin, Charité – University Medical Center Berlin, Freie Universität Berlin and Humboldt-Universität zu Berlin, Berlin, Germany; 8 Institute of Biometry and Clinical Epidemiology, Charité – University Medical Center Berlin, Berlin, Germany; 9 German Center for Child and Adolescent Health (DZKJ), Partner Site Berlin, Berlin, Germany

**Keywords:** hydroxyurea, low-resource settings, real-word data, sickle cell disease, Sub-Saharan Africa

## Abstract

**Background:**

Sickle Cell Disease (SCD) causes acute and chronic complications. Hydroxyurea (HU) reduces vaso-occlusive crises (VOCs) and improves patients’ outcomes. However, evidence on the real-world implementation and effectiveness of HU in resource-limited primary care settings, particularly those serving informal settlements, remains scarce. This study evaluates HU use and its effects in two Nairobi clinics serving informal settlements.

**Methods:**

We retrospectively analyzed 2,206 clinical visits from 328 SCD (HbSS) patients (March 2019–March 2021), comparing complication rates in HU users and non-users. Linear mixed and competing-risk Cox regression models assessed HU’s effects on laboratory markers and clinical outcomes.

**Results:**

HU users had lower incidence rates of VOCs (19.6 vs. 24.7 per 10 person-years) and infections (11.3 vs. 12.4) than non-users. Adjusted analyses showed HU increased hemoglobin (+0.36 g/dL, 95% CI: 0.19 to 0.53) and MCV (+3.47 fl, CI: 2.20 to 4.73). HU reduced the hazard of VOC (HR = 0.82, 95% CI: 0.66 to 1.02) and infections (0.72, 0.55 to 0.95), but evidence for an effect on major complications was inconclusive due to substantial imprecision (1.39, 0.40 to 4.78). Lower HU doses (<20 mg/kg/day) had similar hazard of VOCs as higher doses (20–25 mg/kg/day), but reduced infection and major complication risks.

**Conclusion:**

HU reduces infections and VOCs, but does not relevantly impact major complications. Long-term studies are needed to evaluate its effectiveness in low-resource settings, where adherence is influenced by accessibility and socio-economic factors.

## Background

Sickle cell disease (SCD) comprises a group of inherited monogenic hemoglobinopathies caused by mutations in the *β*-globin gene, characterized by the presence of hemoglobin S (HbS) in combination with homozygous or compound heterozygous genotypes, including HbSS, HbSC, and HbSβ-thalassemia.

HbSS is the most frequent and clinically severe subtype of sickle cell anemia (1). The presence of this abnormal hemoglobin induces vaso-occlusions, vasculopathy, and chronic hemolysis, resulting in both acute and chronic complications (2, 3). There are approximately 510,000 children with SCD born every year worldwide (4) and Sub-Saharan Africa (SSA) countries account for the majority of them (5). In Eastern Africa the homozygous genotype (HbSS) is predominant and Kenya records an estimated number of 14,000 newborns with hemoglobinopathies each year (6). SCD remains a major but neglected public health issue in low-income countries (4), especially in Africa, where under-five mortality rates reach 50–90% (7).

Hydroxyurea (HU) is the only widely licensed disease-modifying therapy for SCD and is considered suitable for broad use in low-resource settings due to its oral formulation and favorable bioavailability (8). Its benefits include increasing fetal hemoglobin, raising hemoglobin (Hb) levels and mean corpuscular volume (MCV), and reducing anemia severity and inflammation (9), though it remains a mild cytotoxic and myelosuppressive agent (10), as the dose used is several-fold lower than that used for oncological treatment.

HU was licensed for pediatric use after its initial approval in adults in 2017, after studies confirmed its benefits in children (11–14), including African regions burdened by infections and malnutrition (15, 16). However, socio-economic and cultural barriers limit access and adherence, leaving many SCD patients untreated or suboptimally managed. HU dose escalation to the maximum tolerated dose has consistently demonstrated superior clinical efficacy and favorable safety profiles in controlled trials, including studies conducted in SSA (17–20). However, the successful implementation of dose-escalation strategies depends on regular laboratory monitoring, frequent clinical follow-up, and reliable treatment continuity. These requirements may be difficult to sustain in underserved, low-resource primary-care settings, where diagnostic capacity and longitudinal follow-up are often limited. Although trials such as REACH (21) and NOHARM (15) provide strong evidence for the effectiveness and safety of HU under research-supported conditions, an important implementation gap remains regarding how these regimens can be optimally delivered and monitored in environments that lack comparable infrastructure and resources (22).

Unlike previous HU trials in Africa, conducted within public tertiary hospitals supported by external research funding, our study focuses on children with SCD living in informal urban settlements in Nairobi. These conditions reflect the broader realities across much of SSA, where HU use remains inconsistent despite national guideline endorsement, largely due to constraints in affordability, drug availability, and monitoring capacity. Pediatric HU formulations are rarely available, requiring caregivers to prepare doses at home, while routine hematological monitoring for safety and dose optimization is limited by restricted laboratory infrastructure and irregular follow-up, despite recommendations for clinical review every three to a maximum of 4 months. These barriers are compounded by structural determinants common to low-resource settings – including overcrowding, inadequate housing, limited access to water and sanitation, economic instability, transportation challenges, and fragmented primary-care services, which collectively shape health-seeking behavior, treatment adherence, and prescribing practices. The aim of this study was to evaluate the real-world effectiveness of HU among children with SCD receiving care in informal settlements in Nairobi, complementing findings from research-supported trials and informing strategies to expand HU access and decentralize SCD care in Kenya and similar settings.

## Methods

### Study design

This retrospective study reviewed patient medical records from March 1, 2019, to March 31, 2021, in two centers of Nairobi, Kenya: Ruaraka Uhai Neema level 4 Hospital (~180,000 residents, from the North-East area of Nairobi) and Baraka Health Center, located in Mathare informal settlement (~500,000 residents, surrounding Ruaraka). Systematic sampling was applied to the registry of approximately 1,000 patients: after choosing a random start, every second patient on the ordered list was selected, resulting in a sample of 500.

#### Inclusion criteria

They were designed to ensure adequate diagnostic confirmation, sufficient clinical documentation, and reliable follow-up data for the assessment of longitudinal HU use and hematologic response. Patients were eligible for *inclusion* if they had (i) a positive sickling test used as an initial screening tool and (ii) a confirmed diagnosis of SCD by hemoglobin electrophoresis high-performance liquid chromatography (HPLC), performed at the study clinic or at an external accredited laboratory. Eligible patients were also required to have sufficiently complete medical records, including basic demographic information (e.g., sex, age), visit date and type (emergency or routine), documented clinical assessment, and prescribed medications.

#### Exclusion criteria

Patients were excluded if they had inherited hematological conditions known to independently affect hemoglobin levels or hemolysis (e.g., *β*-thalassemia intermedia/major, HbS/β-thalassemia, or thrombophilic disorders). Differentiation between HbSS and HbSβ^0^-thalassemia was based on hemoglobin electrophoresis patterns with HbA₂ quantification using HPLC. Thrombophilia was defined by a documented personal history of thrombotic events, a prior clinician diagnosis, where available, or confirmatory coagulation investigations. Patients were also excluded if medical records contained substantial missing or inconsistent demographic information (e.g., unclear patient identification or contradictory demographic data), illegible or incomplete laboratory reports, or insufficient documentation of symptoms, diagnoses, or treatment changes. These exclusions ensured that the analytic cohort consisted of patients whose clinical course and HU exposure could be reliably reconstructed.

#### Definition of HU exposure and follow-up

To enable meaningful longitudinal analyses of HU exposure and treatment response, only “active” patients were included, defined as those with at least two documented scheduled clinical encounters occurring at least 2 months apart during the study period. This criterion allowed identification of HU initiation, treatment continuity, and follow-up patterns while excluding isolated consultations or emergency-only encounters.

The final analytic cohort comprised 328 patients.

The HU was prescribed in our population at a fixed-dose, not with MTD. For a subset analysis, specific HU exposure windows were defined where patients were considered *HU users* after receiving HU continuously for at least a 3-month post-initiation period during which they had received a stable minimum dose of 20 mg/kg/day, while *non-users* were defined as such after a 6-week post-discontinuation period during which no HU was used. These cut-offs were selected to reflect routine follow-up and the expected timing of HU effects. A 3-month exposure period was considered the minimum duration required to observe meaningful hematological and clinical responses, consistent with previous literature (23, 24). Using 6 weeks for the washout period was a pharmacologically informed choice to allow for the expected decline of HU-induced treatment effects after discontinuation.

This resulted in the classification of 274 patients as *HU users* or *non-users*.

The ethical approval of the study was granted by the Kenya Medical Research Institute (KEMRI) Scientific and Ethics Review Unit (SERU): Protocol no. KEMRI/SERU/CIPDCR/060/4985.

### Data management

After a trained consultant pediatrician retrieved medical records from the outpatient clinic’s paper files, data on pre-specified variables were extracted and entered into *Climedo* software (*Climedo Health GmbH,* web application). Chart abstraction was performed electronically and stored in *Climedo*. The collected data were anonymized, encoded, and exported for analysis using a database management system.

If patients were not admitted to Ruaraka Uhai Neema Hospital, relevant data were sourced from the paper discharge summaries; in addition, all outpatient data were collected from the two study clinics, where routine follow-up visits were scheduled every three to a maximum of 4 months.

The study does not involve any contact or collection of data directly from patients. The access of patients’ medical data through clinical encounter forms from outpatient clinics and inpatient information was done in the respect of privacy and confidentiality. The data collected were de-identified and de-linked from the patients’ names.

Basic information were collected for all patients and included age, sex, study visit date, and child health indicators as anthropometric measurements—weight, height or length, weight-for-age ratio, underweight defined as weight below the 5th CDC reference percentiles (25), clinical parameters and laboratory parameters [Hb (g/dl), hematocrit (%)], MCV (mean corpuscular volume, fl), platelets (x10^3/mL), white blood cell count, absolute neutrophil count, and treatments (HU use and dosage, Penicillin V, folic acid, zinc).

To evaluate the association between specific HU dosages and outcome parameters, we defined the following categories for HU dosage: less than 20 mg/kg/day, 20–25 mg/kg/day, 25–35 mg/kg/day.

Laboratory parameters used as outcome parameters in this study were Hb (g/dl) and MCV (fl), and the clinical parameters were vaso-occlusive crisis (VOC), infections, and major complication. These outcomes were selected because they represent clinically relevant indicators of HU effectiveness, are routinely monitored in the care of patients with SCD, and were consistently documented. VOC was defined as any painful episode documented by clinicians, including any kind and severity of pain occurring either at home or in healthcare settings and requiring analgesic treatment for more than 1 day. Infections were identified based on the treating clinician’s documented diagnosis in the medical records and reflected routine clinical practice in these settings. Laboratory or microbiological confirmation was not consistently available and therefore was not required for outcome classification. Infections included any episode of upper respiratory tract infection, pneumonia, viral-induced acute bronchospasm, malaria, gastroenteritis, and osteomyelitis, based on symptom assessment and physical examination rather than pathogen-specific testing. Major complications included acute chest syndrome, aplastic crisis, splenic sequestration, stroke, leg ulcers, and previous history of stroke or leg ulcers with persistent sequelae. These complications were selected because they were consistently documented in the medical records and represent established severe complications of SCD reported in international guidelines and the literature (26, 27).

### Statistical analysis

For description, categorical variables are presented as absolute and relative frequencies, continuous variables with median and interquartile range (IQR). We present patient characteristics at the first study visit, where the unit of observation is the patient, stratified by prior HU use. Given the retrospective analysis of longitudinal patient data, we used the individual patient-visit as the unit of observation for the longitudinal parameters, including the outcome parameters. Incidence rates of clinical outcomes in the subset of HU users and non-users are derived per 10 person-years, along with 95% confidence intervals (CI).

The association of HU use and its dosage with the laboratory measurements of Hb and MCV is analyzed based on a mixed linear regression, with a random intercept per patient. To account for confounding, we adjusted these analyses for age (at the prior visit), sex, underweight, the respective laboratory measurement at the prior visit (serving as a baseline), and major complications in the anamnesis. All time-to-event analyses relating HU use and dosage to the three clinical outcomes are based on an Anderson-Gil competing-risk Cox regression, where death was the competing event, with a frailty term as a random effect per patient. These models were adjusted for sex, age at the visit, and prior complication (VOC, infection, or major complication, respectively). The respective effect estimates are displayed as regression coefficients and hazard ratio (HR) estimates, along with 95% CIs.

Data curation and analysis was carried out with R, just as additional R packages (28–30).

## Results

In total, 328 children were enrolled in this study with a total of 2,206 patient-visits. Among those, about half were female (49.7%). The median age at study entry was 8.3 years (IQR: 3.7–12.2), median age at diagnosis 2.1 years (1.0–4.6), median follow-up 1.2 years. At study entry, 221 (67.4%) attended the SCD clinic at Baraka Health Center, while 107 (32.6%) were seen at Ruaraka Uhai Neema Hospital. Among the female patients, 26 were of reproductive age (15–49 years), of whom 5 had already one child (Table 1). Baseline characteristics were similar across study sites with respect to sex (Baraka Health Center: 49.8% female; Ruaraka Uhai Neema Hospital: 51.4% female) and median age at study entry (8.6 vs. 8.0 years), while median age at diagnosis was slightly lower at Ruaraka Uhai Neema Hospital (1.5 vs. 2.5 years).

**Table 1:** Patient-level demographic and clinical history characteristics, stratified by prior use of hydroxyurea.

	No HU use prior to first visit (*n* = 208)	HU use prior to first visit (*n* = 120)	Total (*n* = 328)
Gender			
Female	109 (52.4%)	56 (46.7%)	165 (50.3%)
Male	99 (47.6%)	64 (53.3%)	163 (49.7%)
Age at study entry (years)			
Median (IQR)	6.3 (2.1, 11.3)	10.2 (6.2, 14.0)	8.3 (3.7, 12.2)
Age at diagnosis (years)			
Median (IQR)	2.1 (1.0, 4.9)	2.1 (0.9, 4.1)	2.1 (1.0, 4.6)
Missing	57	45	102
Follow-up time per patient (years)			
Median (IQR)	1.1 (0.2, 1.8)	1.4 (0.3, 1.9)	1.2 (0.2, 1.8)
Age at study end (years)			
Median (IQR)	8.4 (4.2, 13.4)	12.2 (8.2, 16.1)	10.4 (5.8, 14.3)
Total number of visits within study period			
Median (IQR)	6.0 (3.0, 10.0)	7.0 (3.0, 10.0)	7.0 (3.0, 10.0)
Study site at study entry			
Baraka	149 (71.6%)	72 (60.0%)	221 (67.4%)
Ruaraka Uhai Neema	59 (28.4%)	48 (40.0%)	107 (32.6%)
Parity at study entry			
0 + 0	7 (3.4%)	13 (10.8%)	20 (6.1%)
0 + 1	1 (0.5%)	0 (0.0%)	1 (0.3%)
1 + 0	4 (1.9%)	0 (0.0%)	4 (1.2%)
1 + 1	1 (0.5%)	0 (0.0%)	1 (0.3%)
Not applicable	195 (93.8%)	107 (89.2%)	302 (92.1%)
Complications at study entry			
Transfusions	107 (51.4%)	103 (85.8%)	210 (64.0%)
>10 transfusions	2 (1.0%)	5 (4.2%)	7 (2.1%)
Acute chest syndrome	1 (0.5%)	2 (1.7%)	3 (0.9%)
Asthma	3 (1.4%)	5 (4.2%)	8 (2.4%)
Avascular necrosis	0 (0.0%)	3 (2.5%)	3 (0.9%)
Eyes	1 (0.5%)	0 (0.0%)	1 (0.3%)
Priapism	3 (1.4%)	3 (2.5%)	6 (1.8%)
Kidney	2 (1.0%)	0 (0.0%)	2 (0.6%)
Stroke	8 (3.8%)	10 (8.3%)	18 (5.5%)
Ulcers	2 (1.0%)	1 (0.8%)	3 (0.9%)
Splenic sequestration	3 (1.4%)	1 (0.8%)	4 (1.2%)
Aplastic crisis	0 (0.0%)	0 (0.0%)	0 (0.0%)

Those who used HU prior to the first visit were older at study entry but had a similar age at diagnosis, indicating HU was initiated not at disease onset but in response to disease progression, as evidenced by higher rates of prior transfusions and more than 10 transfusions, despite low overall complication rates. HU, hydroxyurea. IQR, interquartile range.

120 (36.6%) of all patients commenced HU prior to their first study visit and used it during the study period, somewhat more frequently at Ruaraka Uhai Neema Hospital (44.9% vs. 32.6%). Those who used HU were on average about 4 years older and had a similar age at diagnosis compared to those who did not use HU prior to the first visit, indicating that HU use was not driven by disease severity at onset, but rather by disease progression over time. They more often reported a history of prior transfusions (85.8% vs. 51.4%), and had more frequently received more than 10 transfusions (4.2% vs. 1.0%), suggesting that HU was typically initiated in patients with more severe or chronic disease. Complications were low among both groups, with marginally more prior incidences of stroke, priapism, avascular necrosis, asthma, and acute chest syndrome reported among those who used HU prior to the first visit.

Out of 2,206 total visits, patients had varying HU dosages, with 45.9% of visits having no prior HU treatment, 17.8% had dosages below 20 mg/kg/day, 34.9% fell within 20–25 mg/kg/day. Only and a smaller proportion (1.5%) received 25–35 mg/kg/day, as treatment usually followed routine fixed-dose practice, especially due to monitoring limitations typical of the study setting. The median patient weight showed variation according to HU dosage, with the highest weights observed in the 20–25 mg/kg/day group. The high proportion of 39.2% of visits documenting patients with underweight may reflect malnutrition or other health challenges within the population. Regarding complications, VOCs, major complications, and infections were more frequently documented in patients without previous HU use. Hb and MCV levels were higher among those who previously received HU, and additionally in tendency higher among those receiving higher doses (Table 2).

**Table 2:** Visit-level longitudinal clinical characteristics, stratified by prior hydroxyurea use.

Variables	NO HU treatment prior visit (*n* = 1,012)	HU before visits <20 mg/kg/day (*n* = 392)	HU before visit 20–25 mg/kg/day (*n* = 770)	HU before visit 25–35 mg/kg/day (*n* = 32)	Total (*n* = 2,206)
Weight <5th percentile					
Yes	402 (40.3%)	152 (39.0%)	281 (37.1%)	17 (54.8%)	852 (39.2%)
Missing	15	2	13	1	31
Age at the visit (years)					
Median (IQR)	7.96 (3.38, 11.97)	12.87 (9.46, 15.73)	9.68 (6.57, 13.13)	11.22 (7.16, 14.36)	9.82 (5.30, 13.47)
Weight/age					
Median (IQR)	2.99 (2.44, 4.23)	2.79 (2.44, 3.22)	2.82 (2.41, 3.40)	2.34 (2.25, 2.72)	2.87 (2.42, 3.56)
Missing	15	2	13	1	31
Penicillin V					
Yes	841 (83.4%)	225 (57.5%)	617 (80.4%)	18 (56.2%)	1701 (77.4%)
Missing	4	1	3	0	8
Zinc					
Yes	922 (91.5%)	376 (96.2%)	750 (97.7%)	32 (100.0%)	2080 (94.6%)
Missing	4	1	2	0	7
Hemoglobin					
Median (IQR)	7.90 (7.10, 8.60)	8.25 (7.50, 9.30)	8.20 (7.50, 9.10)	8.65 (8.07, 9.43)	8.10 (7.30, 9.00)
Missing	259	94	156	8	517
MCV					
Median (IQR)	77.20 (70.57, 83.82)	81.60 (74.05, 89.20)	86.25 (78.73, 94.57)	88.60 (79.12, 96.08)	81.10 (73.60, 89.60)
Missing	288	109	184	8	589
WBC					
Median (IQR)	15700 (12050, 20070)	13100 (9950, 16400)	12,600 (9300, 16100)	12715 (4925, 16675)	13900 (10440, 17900)
Missing	273	101	171	8	553
Neutrophils					
Median (IQR)	6700 (4700, 9700)	5800 (3903, 7900)	5,800 (3885, 8485)	4695 (2525, 7268)	6200 (4200, 8955)
Missing	283	106	183	10	582
Platelets					
Median (IQR)	354000 (254000, 460000)	374000 (273250, 463250)	356000 (247000, 44400)	422500 (306500, 481750)	360000 (254000, 457000)
Missing	286	102	171	10	569
VOC					
Yes	398 (39.3%)	127 (32.4%)	219 (28.4%)	4 (12.5%)	748 (33.9%)
Major complication					
Yes	16 (1.6%)	3 (0.8%)	8 (1.0%)	0 (0.0%)	27 (1.2%)
Infection					
Yes	215 (21.2%)	48 (12.2%)	124 (16.1%)	3 (9.4%)	390 (17.7%)

HU use was predominantly low-dose, with most patients receiving moderate doses (20–25 mg/kg/day), and higher doses were rare, reflecting routine fixed-dose practice. Patients on HU had higher hemoglobin and MCV, lower frequencies of vaso-occlusive crises and complications, suggesting a beneficial effect of treatment. HU, hydroxyurea. IQR, interquartile range. MCV, mean corpuscular volume. VOC, vaso-occlusive crisis. WBC, white blood cells.

**Figure 1:**
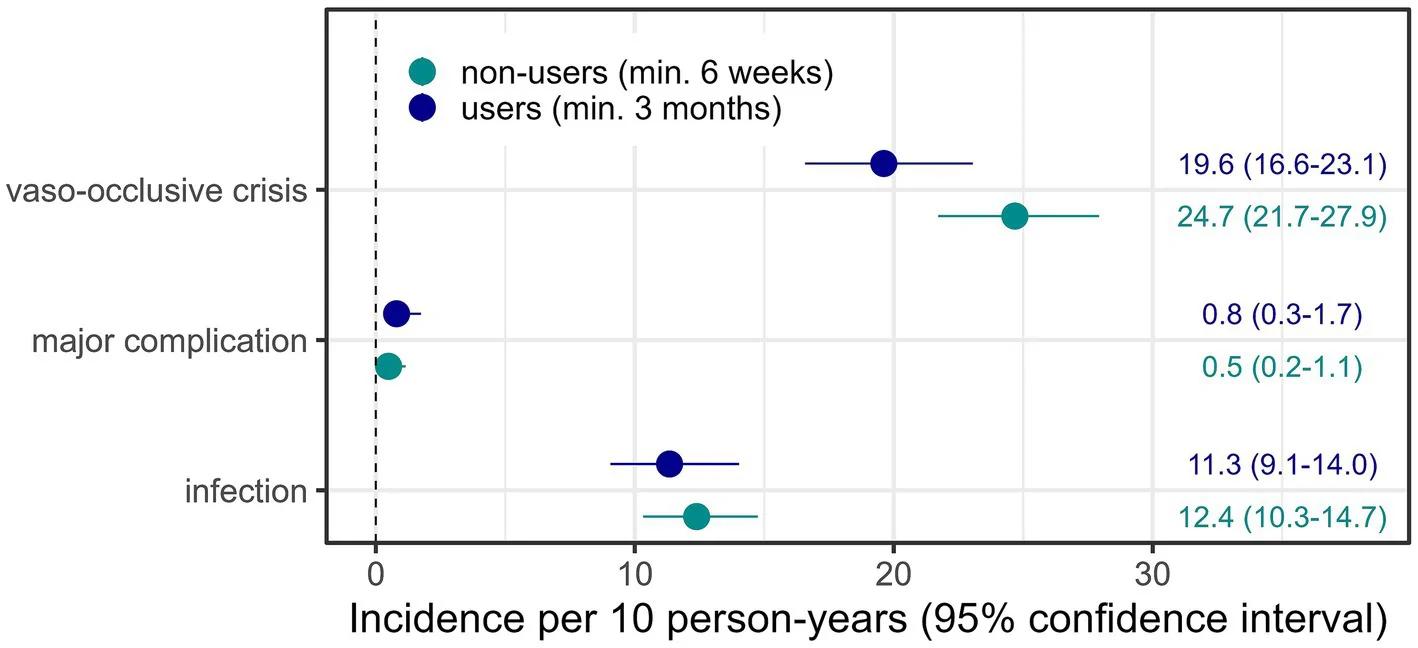
Incidence rates of clinical outcomes per 10 person-years comparing users and non-users of hydroxyurea. Non-users (green) had higher incidences of VOCs compared to users (blue). Whereas no major differences were seen infection rates and incidences of major complications.

Based on the subset analysis for specific HU exposure groups, the incidence rate of VOCs and infections were higher in those not using HU for at least 6 weeks, compared to those who used HU with a dosage ≥20 mg/kg/day for at least 3 months. Per 10 person-years, the VOC incidence rate was somewhat higher among HU non-users (24.7, 95% CI: 21.7 to 27.9, vs.19.6, 95% CI: 16.6 to 23.1), just as the infection rates (12.4, 95% CI: 10.3–14.7 vs. 11.3, 95% CI: 9.1–14.0). The incidence of major complications within this study population was overall low, with the slight trend of more complications within HU users (0.8, 95% CI: 0.3 to 1.7) compared to non-users (0.4, 95% CI: 0.2 to 1.1) (Figure 1). Incidence rates stratified by study site showed similar differences by HU exposure group, but somewhat higher rates of major complications and infections at Ruaraka Uhai Neema Hospital.

**Figure 2:**
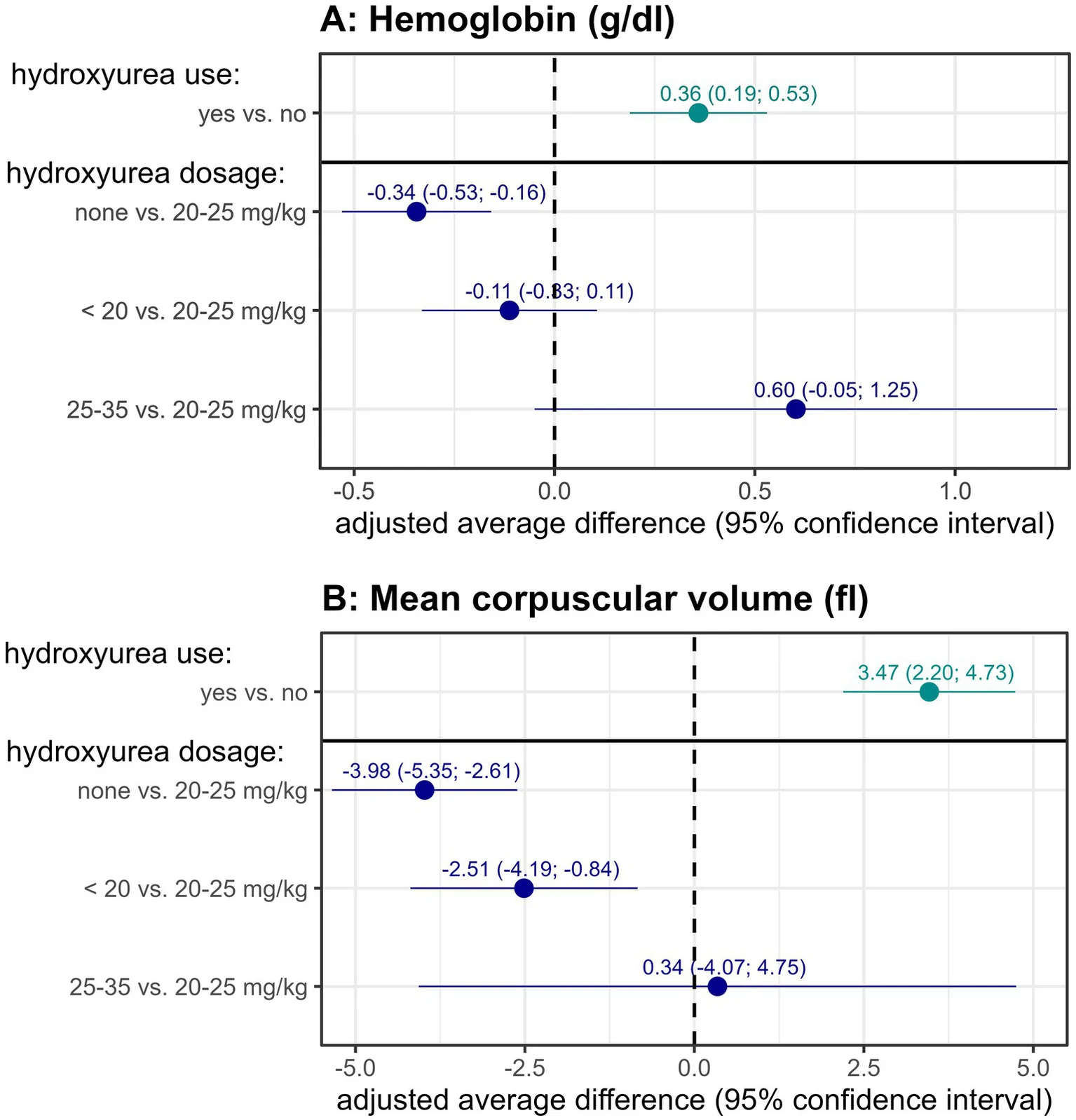
Adjusted average differences showing the association between hydroxyurea use and dosage and **(A)** hemoglobin, **(B)** MCV (regression coefficients are derived from mixed linear regression models, presented along with 95% confidence intervals).

Both laboratory outcome parameters, Hb and MCV, are increased in those reporting prior use of HU. Specifically, the use of HU prior to the respective study visit increases the Hb level by on average 0.36 g/dL (95% CI: 0.19 to 0.53), and the MCV level by 3.47 fl (95% CI: 2.20 to 4.73) (Figure 2A). Although dosages below 20 mg/kg/day and those in the 20–25 mg/kg/day range showed similar associations with Hb levels (−0.11, 95% CI: −0.33 to 0.11), dosages above 20 mg/kg/day were more clearly associated with improved Hb outcomes (Figure 2A). Similarly, higher HU dosage was associated with increased MCV levels, with a clinically relevant difference emerging for dosages below 20 mg/kg/day, which resulted in notably lower MCV compared to the 20–25 mg/kg/day range (−2.51, 95% CI: −4.19 to −0.84). In contrast, the effects on MCV for dosages between 20 and 25 mg/kg/day and 25–35 mg/kg/day were largely indistinguishable (0.34, 95% CI: −4.07 to 4.75; Figure 2B). Despite the absence of formal adherence metrics, the gradual increase in Hb and MCV are consistent with expected biological response to HU and suggestive of reasonable treatment compliance.

**Figure 3:**
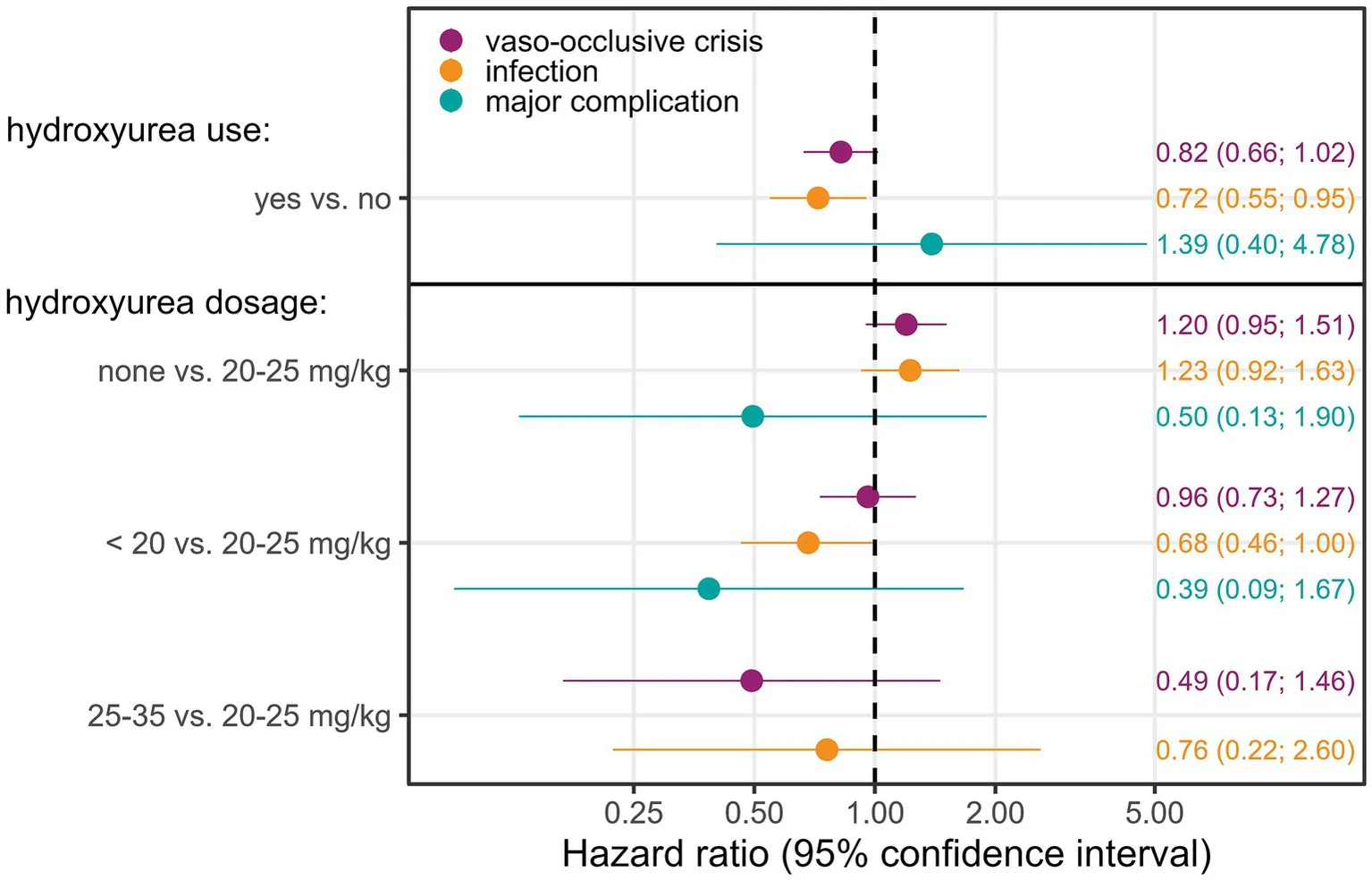
Hazard ratio estimates showing the association between hydroxyurea use and dosage with clinical outcomes (vaso-occlusive crisis, infection, major complication) (hazard ratio estimates are derived from Anderson-Gil competing-risk Cox regression models, where death was the competing event presented along with 95% confidence intervals).

Regarding clinical outcomes, HU use was found to independently reduce hazards of VOC and infections, but not major complication. Hazards to experience a VOC were almost 20% lower for HU users compared to non-users (HR = 0.82, 95% CI: 0.66–1.02), the hazards of an infection were almost 30% lower (HR = 0.72, 0.55 to 0.95). Contrary, the use of HU increased the hazards of a major complication by 40%, but with very high uncertainty in the estimation (HR = 1.39, 0.40 to 4.78), so no inference can be drawn from this finding. Findings on HU dosages are not consistently showing that a certain dosage lowers the hazards of all types of complications. A dosage of < 20 mg/kg/day has similar hazards of VOC as the 20–25 mg/kg/day (HR = 0.96, 0.73 to 1.27), but lower hazards of infection (0.68, 0.46 to 1.00). The high dosage of 25–35 mg/kg/day has in tendency lower hazards of VOC and infection compared to 20–25 mg/kg/day, but the findings are rather unclear due to the low number of events in this high-dosage group, resulting in a wide CI (Figure 3).

## Discussion

Our findings highlight both the clinical benefits of hydroxyurea and the persistent challenges related to its accessibility, implementation, and uptake in resource-limited settings, like Nairobi’s informal settlements. HU did not substantially reduce the incidence of major complications, likely reflecting that, within this population, HU was more frequently prescribed to and used by patients with a higher baseline burden of disease and complications. Notably, HU was associated with a lower incidence of VOCs and infections, both critical complications for SCD patients. This suggests that HU can effectively improve the quality of life of patients when accessible and adhered to in low-resource contexts. HU also seemed to positively impact hematological markers, underscoring HU’s potential to improve anemia management in SCD patients. Interestingly, dosage did not alter the risk of VOCs, however, higher dosages were associated with a reduced risk of infections and major complications, consistent with studies in high-income settings (31, 32). Minimal differences were highlighted in the two study sites, with a higher number of HU-users and as well an earlier age at the diagnosis at Ruaraka Uhai Neema Hospital (level 4 hospital), compared to the SCD patients in Baraka Health Center (which has a catchment area of people with major socio-economic challenges).

Optimal HU dosing and adherence are difficult to achieve in low-income settings due to limited laboratory monitoring, socioeconomic constraints, intermittent drug availability, cultural stigma, and a tendency to initiate treatment only after severe complications (33). In our setting, HU was generally prescribed using a fixed-dose approach rather than dose escalation to maximum tolerated dose, which may have attenuated treatment responses compared with those reported in clinical trials. Although direct assessment of HU adherence was not feasible due the retrospective nature of this study, the observed increase in MCV values provides indirect evidence of sustained HU use among most participants. Nevertheless, variable adherence cannot be excluded and may have contributed to the comparably modest treatment effects.

The relatively small increase in Hb levels is unlikely to reflect poor adherence alone and more plausibly reflects the combined influence baseline anemia severity, intercurrent infections, nutritional status, fixed-dose prescribing and challenges of optimizing therapy in a real-world, resource-limited setting. Although the improvement in Hb was modest, it has to be interpreted in the context of routine fixed-dose HU use in a resource-limited setting, which cannot be directly compared to optimized dose-escalation protocols employed in clinical trials. Furthermore, the higher number of visits and longer follow-up observed among patients using HU prior to the first visit likely reflect structured HU monitoring and greater engagement with routine care rather than differences in disease severity alone, and should be considered when interpreting comparisons between HU users and non-users in this and similar studies. In addition, indication bias should be considered when interpreting the findings as HU, which costs approximately USD 5–10 per month and is not universally provided free of charge in Kenya, it was often reserved for patients with more severe disease, potentially underestimating its real-world effectiveness. Importantly, this study was conducted during a period heavily affected by the COVID-19 pandemic and immediately before the nationwide implementation of the updated Kenyan SCD guidelines recommending HU for all children living with SCD from 9 months of age. As such, these findings provide an important baseline assessment of HU use and effectiveness under routine clinical practice prior to broad policy implementation. Moreover, they remain of relevance as the implementation of the new national guidelines is delayed and has so far been rolled out only in a limited number of areas across the country. These factors likely influenced HU access, prescribing practices and treatment uptake.

To minimize exposure misclassification, patients with undocumented treatment interruptions were excluded from the analysis. Future prospective research and longitudinal studies should assess HU outcomes alongside direct measures of adherence, dose intensity and socioeconomic factors, while comparing outcomes before and after implementation of the updated national guidelines. Such analyses are currently underway and will be reported separately, providing important evidence on the real-world impact of nationwide HU scale-up in Kenya.

The widespread use of zinc supplementation (>90% of participants), an inexpensive supportive intervention associated with reduced infections and other SCD-related complications, further illustrates the feasibility of implementing evidence-based supportive therapies in similar resource-limited settings (34).

As the number of individuals living with SCD continues to grow in SSA (5), African health systems face mounting pressure to address the long-term challenges and to prioritize key questions surrounding the management of SCD. While primary healthcare interventions remain essential (27), the absence of widespread implementation of disease-modifying therapies will exacerbate the burden of SCD. Despite its well-documented benefits for both adults and children in high-income countries (35, 36) and HU is increasingly used for patients with SCD in many parts of SSA (37), concerns remain regarding the potential risks and benefits of HU in SSA: there are no established consensus management recommendations to confirm its safety and efficacy in this context. Therefore, it is essential to conduct prospective evaluations in diverse settings to develop region-specific dosing and monitoring guidelines (38), to understand whether the benefits outweigh the risks. Several clinical trials are currently being conducted across various regions of SSA to better understand the long-term effects of a constant use of HU. Among them is the REACH trial (Realizing Effectiveness Across Continents With Hydroxyurea), investigating the safety and efficacy of HU in different countries, including Kenya (21).

Furthermore, there is a clear need for innovative, resource-appropriate strategies to enhance both HU accessibility and the infrastructure necessary for monitoring its effects. In our setting, HU was typically prescribed at a standardized fixed dosage of approximately 20 mg/kg/day (range 15–25 mg/kg/day), reflecting routine clinical practice and the practical constraints of the local healthcare system. Although this approach improves treatment accessibility and feasibility, it may not achieve the optimal hematological response associated with dose escalation to maximum tolerated dose. Future implementation strategies could therefore explore simplified individualized dosing approaches that balance effectiveness with feasibility while minimizing the need for intensive laboratory monitoring. In parallel, innovative and cost-effective monitoring strategies (39, 40), such as community health worker-led follow-ups or point-of-care testing, or digital adherence support could improve treatment adherence, facilitate dose optimization, and enhance long-term clinical outcomes in resource-limited settings.

The main strengths of this study include the use of a relatively large longitudinal real-world dataset comprising repeated patient-level measurements over routine clinical follow-up, allowing assessment of treatment outcomes over time. Furthermore, the study provides evidence from the implementation of HU in primary care facilities serving informal settlements in SSA, a setting that is underrepresented in the current literature and where real-world effectiveness data remain scarce.

Limitations include the relatively short two-year observation period and inclusion of only two centers. Differential data capture must also be considered, as we cannot rule out that patients receiving HU had more frequent clinic visits, leading to greater documentation of milder illnesses and complications. Socioeconomic bias is possible as access to HU may be associated with better overall healthcare access. Because inclusion required sufficiently complete longitudinal records, the study population may not fully be representative of all SCD patients, particularly those with fragmented care or limited follow-up. The retrospective design limited standardized monitoring of dosing, adherence, and continuity of HU supply. These constraints - together with intermittent drug availability, capsule manipulation, and irregular follow-up - likely contributed to the modest hematologic responses compared with those in controlled clinical trials. Nonetheless, caregivers received training to minimize dosing loss during administration, and the consistent trends observed in key hematologic parameters suggest biologically active HU dosing. We acknowledge that these administration and supply challenges may have attenuated treatment effects, underscoring the need for affordable, child-friendly pediatric HU formulations in similar settings. Furthermore, because this study reflects clinical practice before nationwide implementation of the updated Kenyan SCD guidelines, the findings should be interpreted as a baseline evaluation rather than as representative of current national practice. Ongoing longitudinal analyses comparing outcomes before and after guideline implementation will provide a more comprehensive assessment of the long-term impact of expanded HU access and optimized implementation strategies.

## Conclusion

Our findings demonstrate that HU provides clinically meaningful benefits when implemented in routine primary care in a resource-limited setting, including reduced hospitalization rates and fewer VOCs. The relatively modest magnitude of the observed effects should be interpreted as reflecting real-world implementation challenges rather than the biological efficacy of HU, which has been shown to be substantially greater under optimized treatment conditions. Expanding equitable access to HU, alongside strengthened primary healthcare, newborn screening, and improved implementation strategies, should remain a priority for comprehensive SCD care across SSA.

## Data Availability

The raw data supporting the conclusions of this article will be made available by the authors, without undue reservation.

## References

[1] Steinberg MH. Predicting clinical severity in sickle cell anaemia. Br J Haematol. (2005) 129:465–81. doi: 10.1111/j.1365-2141.2005.05411.x, 15877729

[2] Kato GJ Gladwin MT Steinberg MH. Deconstructing sickle cell disease: reappraisal of the role of hemolysis in the development of clinical subphenotypes. Blood Rev. (2007) 21:37–47. doi: 10.1016/j.blre.2006.07.001, 17084951 PMC2048670

[3] Steinberg MH. Pathophysiology of hemoglobin and its disorders. Disord Hemoglobin. (2009):137–8. doi: 10.1017/CBO9780511596582.012

[4] GBD 2021 Sickle Cell Disease Collaborators. Global, regional, and national prevalence and mortality burden of sickle cell disease, 2000-2021: a systematic analysis from the global burden of disease study 2021. Lancet Haematol. (2023) 10:e585–99. doi: 10.1016/S2352-3026(23)00118-7, Erratum in: Lancet Haematol. 2023; 10(8): e574. doi: 10.1016/S2352-3026(23)00215-6 37331373 PMC10390339

[5] Williams TN. "The clinical epidemiology of sickle cell disease in sub-Saharan Africa". In: Sickle Cell Disease in Sub-Saharan Africa.London, UK: Routledge (2024). p. 4–31.

[6] Ministry of Health (MoH), Kenya. Policy Guidelines on Infant Screening of Sickle Cell Disease. Nairobi: Ministry of Health (2023). Available online at: https://www.health.go.ke/sites/default/files/202306/Policy%20guidelines%20on%20infant%20screening%20of%20Sickle%20Cell%20Disease.pdf (Accessed December 14, 2023)

[7] Grosse SD Odame I Atrash HK Amendah DD Piel FB Williams TN. Sickle cell disease in Africa: a neglected cause of early childhood mortality. Am J Prev Med. (2011) 41:S398–405. doi: 10.1016/j.amepre.2011.09.013, 22099364 PMC3708126

[8] Ware RE Despotovic JM Mortier NA Flanagan JM He J Smeltzer MP . Pharmacokinetics, pharmacodynamics, and pharmacogenetics of hydroxyurea treatment for children with sickle cell anemia. Blood. (2011) 118:4985–91. doi: 10.1182/blood-2011-07-364190, 21876119 PMC3208303

[9] Platt OS Orkin SH Dover G Beardsley GP Miller B Nathan DG. Hydroxyurea enhances fetal hemoglobin production in sickle cell anemia. J Clin Invest. (1984) 74:652–6. doi: 10.1172/JCI111464, 6205021 PMC370519

[10] Lewis WH Wright JA. Altered ribonucleotide reductase activity in mammalian tissue culture cells resistant to hydroxyurea. Biochem Biophys Res Commun. (1974) 60:926–33. doi: 10.1016/0006-291X(74)90403-3, 4429567

[11] Ware RE. How I use hydroxyurea to treat young patients with sickle cell anemia. Blood. (2010) 115:5300–11. doi: 10.1182/blood-2009-04-146852, 20223921 PMC2902131

[12] Strouse JJ Lanzkron S Beach MC Haywood C Park H Witkop C . Hydroxyurea for sickle cell disease: a systematic review for efficacy and toxicity in children. Pediatrics. (2008) 122:1332–42. doi: 10.1542/peds.2008-0441, 19047254

[13] Lanzkron S Strouse JJ Wilson R Beach MC Haywood C Park H . Systematic review: hydroxyurea for the treatment of adults with sickle cell disease. Ann Intern Med. (2008) 148:939–55. doi: 10.7326/0003-4819-148-12-200806170-00221, 18458272 PMC3256736

[14] Wong TE Brandow AM Lim W Lottenberg R. Update on the use of hydroxyurea therapy in sickle cell disease. Blood. (2014) 124:3850–7. doi: 10.1182/blood-2014-08-435768, 25287707 PMC4271176

[15] Opoka RO Ndugwa CM Latham TS Lane A Hume HA Kasirye P . Novel use of hydroxyurea in an African region with malaria (NOHARM): a trial for children with sickle cell anemia. Blood. (2017) 130:2585–93. doi: 10.1182/blood-2017-06-788935, 29051184

[16] Tshilolo L Tomlinson G Williams TN Santos B Olupot-Olupot P Lane A . Hydroxyurea for children with sickle cell anemia in sub-Saharan Africa. N Engl J Med. (2019) 380:121–31. doi: 10.1056/NEJMoa1813598, 30501550 PMC6454575

[17] Khan SJ Zaidi SAT Murtaza SF Asif M Kumar V. Advancements in sickle cell disease (SCD) treatment: a review of novel pharmacotherapies and their impact on patient outcomes. Cureus. (2023) 15:e42847–7. doi: 10.7759/cureus.42847, 37664319 PMC10472971

[18] John CC Opoka RO Latham TS Hume HA Nabaggala C Kasirye P . Hydroxyurea dose escalation for sickle cell Anemia in sub-Saharan Africa. N Engl J Med. (2020) 382:2524–33. doi: 10.1056/NEJMoa2000146, 32579813

[19] Power-Hays A Ware RE. Effective use of hydroxyurea for sickle cell anemia in low-resource countries. Curr Opin Hematol. (2020) 27:172–80. doi: 10.1097/MOH.0000000000000582, 32205588 PMC9364839

[20] Arlet, JB., Bernaudin F. Deme-Ly I. . Use of hydroxyurea in French-speaking sub-Saharan Africa. Ann Hematol 104, 937–941 (2025), doi: 10.1007/s00277-024-06180-2, . 39909904 PMC11971222

[21] Aygun B Lane A Smart LR Santos B Tshilolo L Williams TN . Hydroxyurea dose optimisation for children with sickle cell anaemia in sub-Saharan Africa (REACH): extended follow-up of a multicentre, open-label, phase 1/2 trial. Lancet Haematol. (2024) 11:e425–35. doi: 10.1016/S2352-3026(24)00078-4, 38701812 PMC11289976

[22] Tshilolo L Tomlinson G Williams TN Santos B Olupot-Olupot P Lane A Hydroxyurea for sickle cell anemia children in sub-Saharan Africa. Oncol Times. (2019) 41:22–2. doi: 10.1097/01.COT.0000553528.49029.25, 33079766

[23] Ware RE Aygun B. Advances in the use of hydroxyurea. Hematology Am Soc Hematol Educ Program. (2009) 2009:62–9. doi: 10.1182/asheducation-2009.1.62, 20008183

[24] Buchanan G Yawn B Afenyi-Annan A Ballas S Hassell K James A Evidence-Based Management of Sickle Cell Disease: Expert Panel Report, 2014. Pediatrics. (2014) 134:e1775. doi: 10.1542/peds.2014-2986, 25452651

[25] Meyers A Joyce K Coleman SM Cook JT Cutts D Ettinger de Cuba S Health of children classified as underweight by CDC reference but normal by WHO standard. Pediatrics. (2013) 131:X25–e1532. doi: 10.1542/peds.2012-2382d, 23690515

[26] Yawn BP Buchanan GR Afenyi-Annan AN Ballas SK Hassell KL James AH . Management of sickle cell disease: summary of the 2014 evidence-based report by expert panel members. JAMA. (2014) 312:1033–48. doi: 10.1001/jama.2014.10517, 25203083

[27] Rees DC Williams TN Gladwin MT. Sickle-cell disease. Lancet. (2010) 376:2018–31. doi: 10.1016/S0140-6736(10)61029-X 21131035

[28] R Core Team. R: A Language and Environment for Statistical Computing. Vienna (Austria): R Foundation for Statistical Computing (2014).

[29] Therneau TM Grambsch PM. Modeling Survival Data: Extending the Cox Model. New York: Springer-Verlag (2000).

[30] Abbott R Abbott TD Abraham S Acernese F Ackley K Adams C . Open data from the first and second observing runs of advanced LIGO and advanced Virgo. SoftwareX. (2021) 13:100658. doi: 10.1016/j.softx.2021.100658, 38826717

[31] Quarmyne MO Dong W Barry V Theodore R Adisa OA Buchanan I . Hydroxyurea effectiveness in children and adolescents with sickle cell anemia: a large retrospective population-based cohort study blood. Am J Hematol. (2015) 126:2179–9. doi: 10.1182/blood.V126.23.2179.2179, 27761932 PMC5167640

[32] Ferster A Vermylen C Cornu G Buyse M Corazza F Devalck C . Hydroxyurea for treatment of severe sickle cell anemia: a pediatric clinical trial. Blood. (1996) 88:1960–4. doi: 10.1182/blood.V88.6.1960.bloodjournal8861960, 8822914

[33] McGann PT Ware RE. Hydroxyurea therapy for sickle cell anemia. Expert Opin Drug Saf. (2015) 14:1749–58. doi: 10.1517/14740338.2015.1088827, 26366626 PMC5868345

[34] Miranda CT de OF Vermeulen-Serpa KM ACC P Brandão-Neto J de L VSH Figueiredo MS. Zinc in sickle cell disease: a narrative review. J Trace Elem Med Biol. (2022) 72:126980. doi: 10.1016/j.jtemb.2022.126980, 35413496

[35] Moore RD Charache S Terrin ML Barton FB Ballas SK. Cost-effectiveness of hydroxyurea in sickle cell anemia. Am J Hematol. (2000) 64:26–31. doi: 10.1002/(SICI)1096-8652(200005)64:1<26::AID-AJH5>3.0.CO;2-F, 10815784

[36] Wang WC Ware RE Miller ST Iyer RV Casella JF Minniti CP . Hydroxycarbamide in very young children with sickle-cell anaemia: a multicentre, randomised, controlled trial (BABY HUG). Lancet. (2011) 377:1663–72. doi: 10.1016/S0140-6736(11)60355-3, 21571150 PMC3133619

[37] Mulaku M Opiyo N Karumbi J Kitonyi G Thoithi G English M. Evidence review of hydroxyurea for the prevention of sickle cell complications in low-income countries. Arch Dis Child. (2013) 98:908–14. doi: 10.1136/archdischild-2012-302387, 23995076 PMC3812872

[38] Galadanci NA Abdullahi SU Tabari MA Abubakar S Belonwu R Salihu A . Primary stroke prevention in Nigerian children with sickle cell disease (SPIN): challenges of conducting a feasibility trial. Pediatr Blood Amp Cancer. (2014) 62:395–401. doi: 10.1002/pbc.25289, 25399822 PMC4304992

[39] Teigen D Opoka RO Kasirye P Nabaggala C Hume HA Blomberg B . Cost-effectiveness of hydroxyurea for sickle cell Anemia in a low-income African setting: a model-based evaluation of two dosing regimens. Pharmacoeconomics. (2023) 41:1603–15. doi: 10.1007/s40273-023-01294-3, 37462838 PMC10635957

[40] Smart LR Charles M McElhinney KE Dong M Power-Hays A Howard T . Hydroxyurea pharmacokinetics and precision dosing in low-resource settings. Front Mol Biosci. (2023) 10:1130206–6. doi: 10.3389/fmolb.2023.1130206, 37325474 PMC10267409

